# Bacterial Contamination of Platelet Products

**DOI:** 10.3390/microorganisms12020258

**Published:** 2024-01-26

**Authors:** Michael R. Jacobs, Bowen Zhou, Aditi Tayal, Robert W. Maitta

**Affiliations:** Department of Pathology, Case Western Reserve University and University Hospitals Cleveland Medical Center, Cleveland, OH 44106, USA; bowen.zhou2@uhhospitals.org (B.Z.); aditi.tayal@uhhospitals.org (A.T.); robert.maitta@uhhospitals.org (R.W.M.)

**Keywords:** platelet safety, bacterial contamination, risk control strategies

## Abstract

Transfusion of bacterially contaminated platelets, although rare, is still a major cause of mortality and morbidity despite the introduction of many methods to limit this over the past 20 years. The methods used include improved donor skin disinfection, diversion of the first part of donations, use of apheresis platelet units rather than whole-blood derived pools, primary and secondary testing by culture or rapid test, and use of pathogen reduction. Primary culture has been in use the US since 2004, using culture 24 h after collection of volumes of 4–8 mL from apheresis collections and whole-blood derived pools inoculated into aerobic culture bottles, with limited use of secondary testing by culture or rapid test to extend shelf-life from 5 to 7 days. Primary culture was introduced in the UK in 2011 using a “large-volume, delayed sampling” (LVDS) protocol requiring culture 36–48 h after collection of volumes of 16 mL from split apheresis units and whole-blood derived pools, inoculated into aerobic and anaerobic culture bottles (8 mL each), with a shelf-life of 7 days. Pathogen reduction using amotosalen has been in use in Europe since 2002, and was approved for use in the US in 2014. In the US, recent FDA guidance, effective October 2021, recommended several strategies to limit bacterial contamination of platelet products, including pathogen reduction, variants of the UK LVDS method and several two-step strategies, with shelf-life ranging from 3 to 7 days. The issues associated with bacterial contamination and these strategies are discussed in this review.

## 1. Introduction

Platelet transfusions are important for the prevention or treatment of bleeding in patients with thrombocytopenia or impaired platelet function. Until the general availability of platelets, bleeding was a major cause of morbidity and mortality in patients undergoing chemotherapy. Many obstacles prevented the ready availability of platelets for transfusion and many years elapsed before platelet transfusions became routine practice in the treatment of thrombocytopenic patients. Routine platelet transfusions came into use when Murphy, Sayar and Gardner provided evidence in 1970 that platelets could be stored at 22 ± 2 °C for up to 3 days and still maintain their hemostatic function [1]. Platelet concentrates were shown to maintain their hemostatic function provided the storage conditions met some technical requirements for concentrate volume and pH range, as well as agitation of the concentrate during storage. Further improvements, including the availability of improved storage containers, enabled platelets to be stored for up to 5 and subsequently up to 7 days [2].

Platelet concentrates are susceptible to contamination due to storage conditions that promote bacterial growth. The recommended storage temperature of 20–24 °C permits growth of many bacterial species originating from both the human microflora and environmental sources [3]. Growth of many bacterial species is also promoted by the comparably high oxygen supply as well as by continuous mixing in gas-permeable platelet concentrate bags. Frequently employed additives in the storage solution might also serve as additional energy sources for some microorganisms, resulting in a growth advantage [4].

However, with these advances the issues of bacterial contamination of platelet products and transfusion-related sepsis were soon noted by Buchholz et al. in 1971, with 2.4% of whole-blood derived units found to be contaminated with bacteria and resulting in >20% of pools of 8 units being contaminated [5]. A subsequent report by the same group noted that bacterial counts were initially low and increased during storage at 22 °C, with contamination rates of single, whole-blood derived units being 1.4% on initial culture and 6% after storage for >10 days, with platelet concentrates generally transfused within 3 days of storage due to the high contamination rate [6].

Development of a closed system for production of apheresis platelets was described in the 1980s. A study published in 1985 documented storage of 22 apheresis platelet collections in plasma for 8 days with preservation of platelet function [7]. All 22 of these apheresis collections were sterile at time of production, and 9 other apheresis collections were sterile after 8 days. The 1981 licensure of plastic bags for storage of platelets resulted in extension of storage time from 3 to 5 days, and in 1984 it was further extended to 7 days based on platelet functionality [8]. However, in 1986 the Food and Drug Administration (FDA) Blood Products Advisory Committee noted the magnitude of platelet transfusion-associated morbidity and mortality and rolled back the shelf life of platelet concentrates to 5 days [9].

Subsequent milestones in addressing the problem of bacterial contamination of platelet products included a College of American Pathologists standard, effective December 2002, that recommended that laboratories have a method in place to identify bacterial contamination of these products [8]. In the 22nd edition of the AABB Standards for Blood Banks and Transfusion Services a new standard, effective 1 March 2004, was added requiring that that blood banks or transfusion services have methods that both limit and detect bacterial contamination of platelet products. This led to culture of apheresis collections in 2004 and of pre-storage pooled platelets in 2006 [10], and finally to the long-awaited FDA guidance, Bacterial Detection Testing by Blood Collection Establishments and Transfusion Services to Enhance the Safety and Availability of Platelets for Transfusion, which became effective on 1 October 2021 [11].

## 2. Bacteria Associated with Platelet Product Contamination

### 2.1. Bacterial Species Found in Platelet Products and Their Sources

A wide variety of bacterial species have been found in platelet units. The sources of these contaminants are varied and are shown in Table 1 with examples of typical bacterial species associated with each source. In a study of primary culture of 960,470 apheresis units, 63.5% of bacterial contaminants were species associated with donor skin microbiota, 24.5% with mucous membrane microbiota and 12.0% with other sources [12]. In a long-term study of 126,052 platelet product transfusions, 83.1% of the 77 bacterial species present in platelet units at time of transfusion were typical skin microbiota, while 5.2% were associated with mucous membrane microbiota, 5.2% with gastrointestinal malignancy and 6.7% with environmental contaminants [13]. A recent report on the source of polymicrobial contamination of apheresis units resulting in transfusion-transmitted sepsis contaminated with *Acinetobacter calcoaceticus-baumannii* complex, *Staphylococcus saprophyticus* and *Leclercia adecarboxylata* found evidence that the manufacturer of the platelet collection kits used was the most probable source of this contamination [14]. Retrograde contamination of a platelet unit can also occur in patients with bacteremia at the time of receiving a platelet transfusion as documented by a case where *Enterococcus faecium* was isolated from a patient’s blood cultures before and after transfusion, with the same species recovered from the platelet unit remnants after transfusion [15].

Investigation of the sources of *Staphylococcus aureus* contaminating 16 platelet products, 13 apheresis collections and 3 buffy coat pools, in England documented that a strain identical or closely related to each platelet isolate was cultured from the skin or nares of 12 of the 13 apheresis donors and one of the donors in each of the 3 pools [16].

Environmental bacteria can contaminate platelet products during collection and storage, associated with defects in platelet concentrate storage containers. A study of acquired container defect reports to one manufacturer from January 2019 to July 2020 documented 24 instances of leaks due to damage of apheresis platelet bags, a rate of 44 per million distributed containers [17]. Damage consisted of scratches, impressions, and/or piercings that can provide a pathway for contamination that cannot be detected by primary culture or prevented by pathogen reduction. The source of polymicrobial contamination resulting in a fatal septic reaction, associated with a pathogen-reduced apheresis unit that had a leak visible only on pressure testing, was initially thought to be the defect in the bag, but further investigation found that contamination of platelet collection sets at the manufacturing facility was the most probable source of this and other cases of polymicrobial contamination [14,18].

### 2.2. Bacterial Growth Characteristics

**Bacterial count:** Cells such as erythrocytes, leukocytes, platelets and bacteria that are suspended in liquids such as plasma are usually quantitated as cell counts per standard volume, although many publications document bacterial counts as concentrations or titers. Quantitation of bacteria is usually expressed as counts of colony-forming units (CFU) per mL of the product based on quantitative culture, where fixed volumes (typically 0.1 mL) of serial dilutions (typically 10-fold) of a product are plated on agar media and CFU count calculated from dilutions with countable colonies after incubation. Sensitivity of plate culture using serial dilutions of 0.1 mL is around 10^1^ to 10^2^ CFU/mL [19].

**Bacterial growth:** Bacteria in liquid media typically have four growth phases:

(1) Lag phase, where bacteria adapt to growth conditions, remaining viable but not multiplying.

(2) Logarithmic growth phase, where bacteria multiply at a constant doubling time (also referred to as generation time) if appropriate nutrients and atmosphere are present.

(3) Stationary phase occurs when nutrients are exhausted.

(4) Death or decline phase due to lack of nutrients and increases in toxic metabolic products. Death or decline phase can also occur in products such as platelets suspended in plasma that have antibacterial properties, which can kill bacteria in stationary or early log phases.

**Bacterial growth in platelet concentrates:** Platelets, leukocytes, plasma and platelet additive solutions, in addition to providing nutrients and substrates that promote bacterial growth, also have antimicrobial properties that may result in stasis or killing rather than growth of bacteria. Platelets express a wide range of potential bacterial receptors, have the ability to internalize bacteria, are able to release a broad variety of molecules with antimicrobial activity against bacteria and fungi, and are also able to generate reactive oxygen species, bind and internalize microorganisms and participate in antibody-dependent cellular cytotoxicity [20]. In experimental studies of bacterial growth in platelet susupensions, *Bacillus cereus*, *Pseudomonas aeruginosa*, *Klebsiella pneumoniae*, *Serratia marcescens*, and *Staphylococcus aureus* generally grew rapidly, reaching ≥10^5^ CFU/mL by day 4, whereas *Staphylococcus epidermidis* had slower and more varied growth, with 81.3% reaching 10^2^ CFU/mL by day 3 and 95.8% by day 4 [21]. Anaerobic bacteria such as *Cutibacterium* (*Propionibacterium*) *acnes* show slow or no growth under the aerobic storage conditions of platelet concentrates [22]. Occasional atypical strains of bacterial species regarded as strict anaerobes such as *Clostridium perfringens* have grown in platelet concentrates and caused severe septic reactions [23,24].

Lag times of bacteria inoculated into platelet concentrates have been shown to range from 0 to 116 h, with most lag times being <24 h [25]. Lag times depended on inoculum size, with smaller inocula associated with longer and more variable lag times, as well as on variables associated with different bacterial species and the environment of platelet bags. Doubling times in log growth phase of bacterial species associated with contamination of platelet concentrates under storage conditions of platelet concentrates range from 0.8 to 12 h, with a lower 90% range of 1–5 h [25]. Based on these parameters, estimates of median times that a single organism growing in a 350 mL platelet unit will reach 10^5^ CFU/mL, the minimum bacterial load associated with septic transfusion reactions [19], are shown in Table 2. Fast-growth species with short lag times can reach 10^5^ CFU/mL within 2 days, whereas slower growers with longer lag times can take up to 5 days to reach this bacterial load.

## 3. Measures Taken to Decrease the Risk of Bacterial Contamination

Measures taken to limit bacterial contamination of platelet products have evolved over time and include optimizing the skin preparation technique of donors, diversion of the initial 15 to 30 mL of the blood draw, ensuring sterility and integrity of materials and methods used to process blood components, changing supply from whole-blood derived to apheresis units, prepooling and primary culture of whole-blood derived units, primary culture of whole-blood derived pools and apheresis units, secondary culture of apheresis units, rapid test within 24 h of issue of whole-blood derived and apheresis units, and pathogen reduction of apheresis units and whole-blood derived pools [26,27].

**FDA guidance on bacterial contamination risk control strategies to enhance the safety and availability of platelet products.**

The production, use and shelf-life of platelets in the US is set by the Center for Biologics Evaluation and Research (CBER) of the US FDA based on regulations issued in Title 21 of the Code of Federal Regulations [26]. In 1984 FDA increased the shelf life of platelet concentrates from 5 to 7 days, but in 1986 reduced this back to 5 days based on an increase in reports of septic transfusion reactions. Various strategies have been used since 2004 on products or systems cleared by the FDA to limit bacterial contamination, including primary culture, rapid testing and pathogen reduction. FDA published draft guidance in 2014, which was followed by several updates and three FDA Blood Products Advisory Committee Meetings, with guidance finalized in 2020 with an implementation date of 1 October 2021 [11]. This guidance includes multiple strategies for limiting bacterial contamination of platelet products.

Each page of the guidance document has this header: “**Contains Nonbinding Recommendations**” and this statement is included on the box header on page 1: “This guidance represents the current thinking of the Food and Drug Administration (FDA or Agency) on this topic. It does not establish any rights for any person and is not binding on FDA or the public. You can use an alternative approach if it satisfies the requirements of the applicable statutes and regulations. To discuss an alternative approach, contact the FDA staff responsible for this guidance as listed on the title page”. A similar statement under the introduction states:

“FDA’s guidance documents, including this guidance, do not establish legally enforceable responsibilities. Instead, guidances describe the FDA’s current thinking on a topic and should be viewed only as recommendations, unless specific regulatory or statutory requirements are cited. The use of the word *should* in FDA’s guidances means that something is suggested or recommended, but not required”. Despite these caveats, the guidance was implemented by all US blood product suppliers by the implementation date in the guidance.

The FDA guidance provides two broad options:

1. Pathogen reduction of apheresis units using an FDA approved pathogen reduction device according to the device instructions for use, with no need for further measures to control the risk of bacterial contamination. Shelf-life of platelet units treated by FDA approved pathogen reduction devices is currently 5 days.
2. Testing of platelet products by culture and/or rapid test by single- or two-step strategies.
  - Testing strategies for apheresis and pre-storage whole-blood derived pools, shown in Table 3, include three single-step primary culture options and six two-step strategies requiring culture for primary testing and either culture or rapid test for secondary testing. Shelf-life of platelet products ranges from 3 to 7 days depending on the product type and the strategy used.
  - Testing strategies for single and post-storage pooled whole-blood derived units are shown in Table 4. Shelf-life of platelet products ranges from 3 to 5 days depending on the product type and the strategy used.

## 4. Methods for Determining Bacterial Contamination of Platelet Products

While a wide variety of direct and indirect methods have been used to test platelets for the presence of bacterial contamination, only the few that are in current use will be discussed. Tests performed on platelet products have been defined by the recent US FDA guidance document on this subject as primary and secondary [11]. Primary testing is the initial bacterial detection test, usually by culture, performed following collection and before release of products for transfusion. Secondary testing is any additional test to detect bacteria in a platelet unit that showed no bacterial contamination upon primary testing. Secondary testing can be by culture or rapid testing methods.

### 4.1. Culture

#### 4.1.1. Direct Culture

Small volumes of liquids can be inoculated directly onto agar plates, which are then incubated in a variety of atmospheres and temperatures. Direct culture of platelet products is typically performed by inoculating 0.1 mL volumes onto blood agar plates, which are incubated in a 5% CO_2_ atmosphere for up to 48 h. Anaerobic cultures can also be performed by incubating a second plate in an anaerobic atmosphere. The bacterial load of the specimen can be calculated from the number of colonies that grow, and serial 10-fold dilutions of a positive sample can be cultured to determine the bacterial load if the undiluted culture has too many colonies to count accurately. Direct culture is indicated when expected bacterial loads are higher than the sensitivity of direct plate culture (10–100 CFU/mL [19]), for example at time of issue of platelet products.

#### 4.1.2. Enriched Culture

When expected bacterial loads are low, for example close to time of collection of platelet products, higher volumes of the product need to be cultured using broth culture media as is done for blood cultures. Three commercially available systems are currently available for performing enriched cultures using bottles containing suitable culture media and atmospheres for aerobic and anaerobic culture—BacT/ALERT (bioMérieux, Marcy-l’Étoile, France), Bactec (BD Biosciences, San Jose, CA, USA) and VersaTREK (ThermoFisher, Waltham, MA, USA). Automated instruments used to test bottles are: BacT/ALERT 3D and BacT/ALERT VIRTUO, Bactec FX system and VersaTREK Automated Microbial Detection System [28,29]. Culture bottles can be inoculated with up to 10 mL per bottle. Bottles are incubated in instruments at 35 °C and monitored for colorimetric changes in pH sensors in the bottles as a result of CO_2_ produced by growing microorganisms or pressure changes in the headspace secondary to gas consumption or production [30]. Bacterial counts at the time of detection by the three automated culture systems ranged from 10^7^ to 10^10^ CFU/mL, with the majority being 10^8^ to 10^9^ CFU/mL [29].

The entire contents of a platelet unit can be cultured using an enrichment culture method, which is particularly useful in evaluating the efficacy of pathogen reduction [31]. In this study, culture of the entire unit at the end of the experimental pathogen reduction procedure was performed by inoculating multiple pairs of aerobic and anaerobic BacT/ALERT bottles will aliquots of 8 mL per bottle.

Investigation of septic transfusion events by culture may have a low yield in patients receiving antibiotics, which can be overcome using metagenomic next-generation sequencing (mNGS) of blood and platelet product specimens [32]. This method was used to investigate cases of transfusion-related sepsis and resulted in discovery of a novel *Acinetobacter* species in a platelet product that had been treated with photochemical pathogen reduction. Advantages of this culture-independent mNGS method include rapid and quantitative pathogen identification, and determination of the genetic relatedness of the pathogens detected.

Sensitivity of culture bottles is around 1 CFU/mL [19]. A report from a period where 8 mL volumes of platelet collections or pools were cultured using aerobic BacT/ALERT bottles estimated that there are 19 collections with low counts of dormant bacteria that are not readily detected by early BacT/ALERT culture for every confirmed positive contaminated collection detected [33]. The sensitivity of primary culture has been estimated to be 31% [34], highlighting the limitations of primary culture to detect bacterial contamination in platelet concentrates.

### 4.2. Rapid Testing

While many methods for rapid testing have been developed, only one, the PGD*prime* Test (Verax Biomedical, Marlborough, MA, USA) is currently commercially available in some countries [35]. This test is a rapid, lateral-flow, qualitative immunoassay for the detection of aerobic and anaerobic Gram-positive and Gram-negative bacteria and is used on the day of transfusion to extend the storage of leukocyte-reduced apheresis platelets in plasma from 5 to 7 days (see Section 3). The test detects a broad range of pathogenic bacteria at loads of 1.9 × 10^3^ to 2.5 × 10^6^ CFU/mL (Table 5), and its sensitivity and specificity as well as ease-of-use have recently been improved over the original PGD Test [36].

In an earlier study using the original PGD Test, the test detected bacterial contamination in 9 of 27,620 platelet doses (326 per million) released as negative by primary culture [37]. The specificity of the PGD*prime* Test in a study of 3800 platelet components of all US platelet product types (except pathogen-reduced) showed no false-positives (100% specificity, with a lower 1-sided 95% confidence limit of 99.9%) [38]. Use of the test to extend storage from 5 to 7 days has been demonstrated to significantly reduce outdating, with more than 1.4 million PGD devices shipped to users to date without any fatal septic reactions resulting from the transfusion of a PGD-negative platelet product.

### 4.3. Test Interpretations

AABB Bulletin #04-07, Actions Following an Initial Positive Test for Possible Bacterial Contamination of a Platelet Unit, issued in October 2004 and updated in June 2022, provides interpretations of tests used to detect bacterial contamination of platelet products, methods for confirmatory testing of initial positive results and definitions for the interpretation of findings [39]. This bulletin also discusses the management of other co-components associated with the same donation and provides guidance to address when a positive test result is encountered only after transfusion of the unit, and when a recipient develops culture-proven posttransfusion sepsis. In this bulletin, a “test” is defined as any method implemented to detect bacterial contamination of platelet products, whether by a culture-based or non-culture-based method. Tests can be interpreted as initial positive, true positive, false positive, indeterminate or false negative. These interpretations apply in any circumstances, including (1) when the component has not been issued, (2) when the component has been issued and transfused, and (3) when the component has been issued based on a negative initial test and the recipient developed posttransfusion sepsis confirmed by a positive culture.

1. **Initial positive**

Positive or abnormal (out-of-range) initial test. When applied to automated instrument systems monitoring culture bottles, an instrument signal indication positivity by the detection method used by the instrument indicates an initial positive result. When applied to rapid tests, this means that an initial positive result was obtained.

2. **True positive**

Positive on both the initial test and a confirmatory test. The confirmatory test must be culture-based and be performed on a different sample than the culture bottle or sample used for the initial test. The sample source for the confirmatory test is typically the original platelet component, which can be tested by the same culture method used to test the original specimen. A subculture of the initial positive culture bottle is not an adequate sample for this purpose. If a sample is not available for confirmatory testing because the unit has been transfused, posttransfusion sepsis in the recipient verified by positive culture is also defined as a true positive. These definitions imply, although they do not definitively state this, that a true positive requires that the bacterial species cultured from the confirmatory culture or the recipient is the same as the species found in the initial positive culture.

3. **False positive**

A false positive is defined as a positive initial test, negative confirmatory test **and no** clinical or microbiological evidence of posttransfusion sepsis in the recipient. False-positive results may occur for several reasons, including contamination during inoculation and by machine or reading error. All are included under this heading for the purposes of these definitions. There are, however, other reasons for false positives that are not included in the AABB false positive definition; these include (1) the presence of low inocula of bacterial species that do not grow in platelet products such as *Cutibacterium* (*Propionibacterium*) *acnes*, where only some of multiple cultures performed at the same time point may be positive [22], and (2) auto-sterilization of the platelet unit resulting in negative confirmatory cultures. Auto-sterilization has been described with coagulase-negative staphylococci [40] and with *Bordetella holmesii* [41].

4. **Indeterminate**

A positive initial test with either no confirmatory test performed or confirmatory test results that could not be interpreted is interpreted as indeterminate. A negative initial test with no confirmatory test performed and recipient shows evidence of posttransfusion sepsis is also interpreted as indeterminate; in this definition evidence of sepsis is presumed to be clinical as microbiological evidence of sepsis is interpreted as a false negative initial test. Other combinations of component and recipient results in situations where the component has been transfused are also interpreted as indeterminate.

5. **False negative**

A negative initial test but the remaining available sample of the unit is positive by confirmatory test after the component has been transfused to a recipient who develops culture-proven posttransfusion sepsis. The same microorganism should be isolated from the component and the recipient. To the extent possible, other sources of bacteremia (e.g., infected indwelling catheter) should be excluded.

6. **True negative**

As part of an investigation of reported posttransfusion sepsis, the unit tests negative on the initial test and the remaining available sample of the unit is negative by confirmatory test.

In clinical practice, the vast majority platelet units with negative initial tests are released after a holding period and then transfused with no transfusion reaction, with the initial test remaining negative for the shelf-life of the unit. The interpretation of these circumstances based on the above AABB definitions is indeterminate as a negative confirmatory culture is required to meet the definition of a true negative.

## 5. Prevalence of Bacterial Contamination of Different Platelet Products

### 5.1. Primary Culture

**Figure 1:**
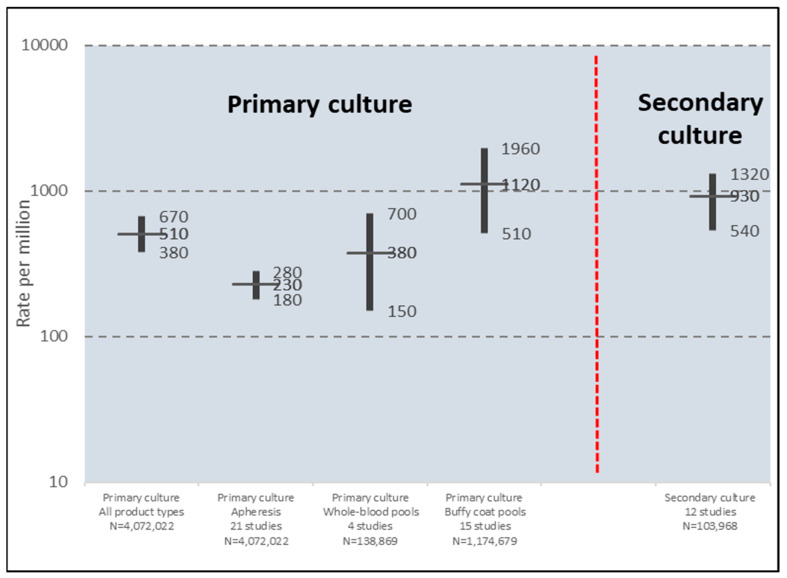
Meta-analysis of 22 studies of bacterial contamination rates of platelet units by primary and secondary culture using a variety of primary and secondary test methods. Plots show point prevalence contamination rates with 95% confidence intervals. Adapted from Walker et al. [34].

Contamination rates of platelet products by primary culture vary considerably due to the variety of sample volumes, bottle types and interpretative criteria used, as well as the limitation imposed by most series having a large proportion of indeterminate findings due to unavailability of transfused products for testing [34]. Nevertheless, a meta-analysis of 22 studies that included 21 apheresis cohorts (4,072,022 components), 4 whole-blood derived pool cohorts (138,869 components), and 15 buffy coat pool cohorts (1,474,679 components) was performed [34]. True positives were defined as bacterial growth on subculture of initial positive bottles in 7 of these studies, or as isolation of the same bacterial species on repeat culture of platelet products in the other 15 studies. Culture volumes generally varied from 4 mL to 20 mL per product, with aerobic only culture bottles used in 9 and both aerobic and anaerobic bottles in 13 studies. Results are shown in Figure 1. The overall mean contamination rate per 1000 components was 0.51 (95% CI 0.38–0.67) (510 per million). The contamination rate was lowest for apheresis units (0.23, 95% CI 0.18–0.28) (230 per million), higher for whole-blood derived pools, (0.38, 95% CI 0.15–0.70) (380 per million), and highest for buffy coat pools (1.12, 95% CI 0.51–1.96) (1120 per million).

A large study of primary culture, designated “large volume, delayed sampling” (LVDS), was performed on 960,470 split apheresis units sampled at 36 to 48 h after donation, with 8 mL inoculated into each of aerobic and anaerobic BacT/ALERT culture bottles, representing 7% of apheresis units by volume [12] illustrates many of the issues associated with primary culture. Using AABB definitions, 3824 tests were initially reactive (3981 per million), with 208 true positives (216 per million; 5.4% of initial positives), 2110 confirmed negatives (2197 per million; 55.2% of initial positives), and 1506 indeterminate (1568 per million; 39.4% of initial positives), (329 indeterminate with positive cultures and 1177 indeterminate with no growth from the initial reactive bottle and platelet units not available for retesting). A summary of the 208 true positive cultures by bacterial species or group, bottle type and time to detection is shown in Table 6. Strict anaerobes that are unable to grow under the aerobic storage conditions in platelet units accounted for 97 of the 208 (46.6%) isolates, representing true positives that are of no clinical significance. Of the 111 (53.4%) that were aerobic or facultative bacteria potentially able to grow and result in septic reactions, 78 were recovered from both aerobic and anaerobic bottles while 37 were recovered from only one of the bottles, indicating low bacterial counts in their source units. Detection times of true positives excluding strict anaerobes ranged from 3 to 42 h, with that of the most virulent species (*Staphylococcus aureus* and *Enterobacterales*) ranging from 3 to 16 h. Overall, this study highlights many of the issues associated with primary culture using both aerobic and anaerobic bottle types, including the low proportion (0.02%; 111/960,470) of true AND clinically significant positives compared to the number of initial positives (0.4%; 3824/960,470), the high proportion of false positive initial reactions (55.2%; 2110/3824) and the high proportion of indeterminates (39.4%; 1506/3824). Encouraging findings from this study include the rapidity of detection of highly virulent pathogens (within 16 h) and the high proportion of true positives with clinically significant pathogens that grew in both bottle types (73.3%; 78/111), suggesting that the delayed sampling (36–48 h) time did allow more contaminating bacteria to reach levels detectable by the culture technique used (16 mL per split apheresis unit).

Another approach to primary testing for bacterial contamination was performed using culture of at least 3.8% by volume of each collection bag, inoculating one to three aerobic BacT/ALERT bottles (7–10 mL per bottle), with sampling from the mother bag 24 to 36 h after collection [34,42,43]. This proportional sample volume method improved the sensitivity of primary testing and was included in initial version of the FDA guidance, but was not included in the final version based in part on the rate of positive outdate cultures of 553 (95% CI 244–1290) per million (5/9041) not being better than low volume strategies [42,43].

### 5.2. Secondary Culture

Meta-analysis of 12 studies using culture as a secondary testing method, with a total of 103,968 primary culture negative components tested, showed a mean positivity rate of 930 (95% CI, 540–1320) per million components, with a wide range of mean rates (220–5270 per million) [34] (Figure 1). Sensitivity of primary culture for detection of contamination, assessed from 9 of the 12 secondary culture studies, was calculated to be 31.1% (95% CI, 22.7–39.5%) based on the mean detection rate by primary culture of 420 (95% CI, 240–600) per million compared to 930 (95% CI, 540–1320) per million by secondary culture based on the combined contamination rates of primary and secondary culture.

### 5.3. Secondary Rapid Test

Meta-analysis of five studies using the PGD rapid test as a secondary testing method, with 114,697 primary culture negative components tested, showed a mean positivity rate of 90 (95% CI, 10–250) per million components, with a wide range of mean rates (0–710 per million) [34]. Six of 14,764 (406 per million) rapid bacterial tests performed in the US in 2021 were positive in a 2021 national survey [44].

## 6. Pathogen Reduction

Three methods for pathogen reduction of platelet concentrates have been developed—INTERCEPT Blood System (Cerus Corporation), Mirasol System (Terumo BCT) and Theraflex UV-C Platelets System (Macopharma) [45]. These systems illuminate platelet concentrate containers with UV light of different wavelengths in the absence (Theraflex UV-C) or presence of a photoactive chemical, amotosalen (INTERCEPT) and riboflavin (Mirasol PRT). INTERCEPT requires post-illumination adsorption to reduce residual amotosalen levels [46]. UV illumination induces irreversible damage of nucleic acids of pathogens, including viruses, bacteria and parasites, as well leukocytes, eliminating the need for gamma irradiation to inactivate leukocytes [45]. While in vitro studies show deleterious effects of pathogen reduction on platelet storage lesions and clinical trials show decreased post-transfusion platelet count in recipients, current evidence suggests that pathogen reduction treated platelet concentrates provide similar protection against clinically relevant bleeding compared to conventional platelet products [45].

**Figure 2:**
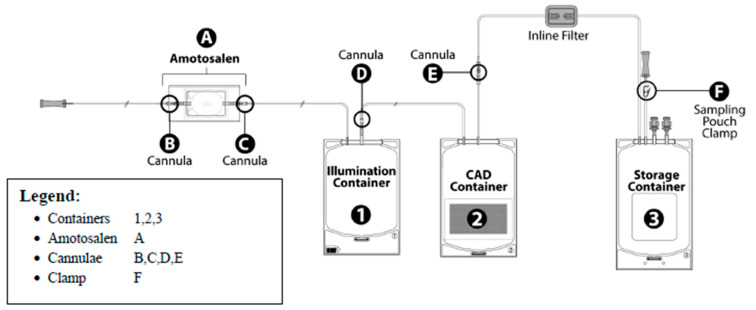
INTERCEPT process for pathogen reduction using small volume processing set [46]. A large volume processing set is also available and includes two illumination containers. CAD, Compound Adsorption Device.

The INTERCEPT pathogen reduction system, illustrated in Figure 2, is complex and includes a pouch containing amotosalen, an illumination container, a Compound Adsorption Device (CAD) to adsorb amotosalen and one or two storage containers. The illumination process takes three minutes and the CAD process takes 4–16 h for Amicus platelet concentrates in PAS-3 or 12–24 h for Trima platelet concentrates in 100% plasma [46]. The INTERCEPT process reduces a broad spectrum of bacteria by >4 log_10_ CFU/mL, but bacterial spores and some viruses (e.g., HAV, HEV, B19 and poliovirus) are resistant to inactivation by the INTERCEPT System. Bacterial inactivation by the INTERCEPT System was tested by adding high inocula of bacteria growing in log phase to platelet units and has not been tested on low inocula of bacteria in lag or early log phase in real-life contaminated platelet products, and systematic study by secondary culture of pathogen reduced platelet units to document sterility have not been performed. Septic reactions have occurred with transfusion of pathogen reduced platelet units (see Section 9).

## 7. Efficacy of Methods for Detection of Bacterial Contamination of Platelet Products

**Figure 3:**
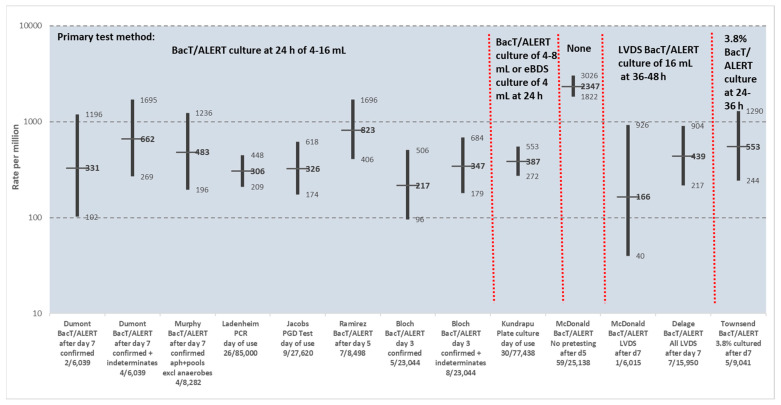
Bacterial contamination rates of platelet units (predominantly apheresis) by secondary testing on day of use or at outdate from multiple studies using a variety of primary and secondary test methods [12,13,43,47]. Plots show point prevalence contamination rates with 95% confidence intervals. Primary test method is shown at the top of the figure. Secondary test method, with details of first author, secondary test method, and results (numerator is number of contaminated platelet units and denominator is number of units tested) is shown on the X-axis. Secondary testing was performed by BacT/ALERT culture (n = 8), PCR (n = 1), PGD test (n = 1), or plate culture (n = 1). Plots show point prevalence contamination rates with 95% confidence intervals (CIs), with results excluding and including indeterminate positives also shown for two of the studies. Point prevalence contamination rates for these data sets where primary testing was used ranged from 166 to 823 per million units, with overlapping 95% CIs for all data sets. The single data set where no primary testing was used had a point prevalence contamination rate of 2347 per million, with 95% CIs not overlapping any of the other data sets. Red dotted lines separate primary test methods.

The efficacy of primary culture has been evaluated based on studies using a variety of primary and secondary test methods on apheresis platelet products for bacterial contamination on day of use or at outdate [12,13,43,47]. Secondary testing was performed by BacT/ALERT culture (n = 8), PCR (n = 1), rapid test (n = 1), or plate culture (n = 1). Results of these studies are shown in Figure 3, with results excluding and including indeterminate positives shown for two of the studies. Primary culture method in 7 of these studies was culture of 4–16 mL at 24 h after collection using BacT/ALERT bottles. No primary testing was performed in one study. Enhanced primary testing was performed in two studies, one using LVDS and the other culture of 3.8% of apheresis collections. The single data set where no primary testing was used had a point prevalence contamination rate of 2347 per million, with 95% CIs not overlapping the CIs of any of the other data sets. Point prevalence contamination rates where primary testing was used ranged from 166 to 823 per million units, with overlapping 95% CIs for all these data sets.

The PASSPORT study was one of the studies shown in Figure 3 and deserves a more detailed description [48]. It was conducted 2005–2008 on apheresis platelet units with an outdate of 7 days (outdate was 5 days at the time the study was performed) under a very specific criterion for success set by the FDA, with 52 participating blood collection centers specially registered or licensed by FDA for 7-day apheresis platelet collections. Primary culture was performed on all collections and secondary culture on all outdated units. Culture was by inoculation of 8–10 mL divided between aerobic and anaerobic BacT/ALERT culture bottles. Primary culture was done 24 to 36 h post-collection and held for 24 h before release of platelet products if negative at that time. A total of 388,903 apheresis collections were accrued with 14 septic transfusion reactions reported, a rate of 36 per million (95% CI 22–60 per million). Secondary culture was performed on 6039 outdated units. The study hypothesis was that the residual risk of detectable bacteria in apheresis platelet products stored for 7 days is less than the risk of untested apheresis platelet products using a 95% confidence limit. Specifically, the criterion for success was that the upper limit of the 95% CI of secondary testing is lower than the combined rates of primary and secondary testing. The primary test positivity rate found in the study was 231 per million (90/388,903 collections) and secondary test rate was 662 (95% CI 180–1695) per million (4/6039 units), with a combined rate of 893 (231 + 662) per million. As the upper limit of the 95% CI of the secondary test (1695 per million) was higher than the combined rate (893 per million) the study was deemed a failure. The sensitivity of primary culture was 25.9% (231/893 per million). Two of the four positive secondary test results had discordant bacterial identifications; if these are considered false positives then its sensitivity increases to 41.1% (231/562 per million). The PASSPORT study had a targeted secondary culture target of 50,000 units and was terminated early with only 6039 tested due to the 4 positive secondary cultures, resulting in a severely underpowered study. Nevertheless, the study was performed with a clear criterion for success set by the FDA based on secondary culture, a principle that has not been applied to subsequent strategies included in the recent FDA guidance document, as was pointed out at the FDA CBER Blood Products Advisory Committee Meeting in 2018 [49].

While the efficacy of primary culture not using enhanced primary testing has been well-established as discussed above, the efficacy of newer strategies, LVDS and pathogen reduction, have not been extensively studied by secondary testing, with their evidence of efficacy predominantly based on rates of reported septic reactions, which has major limitations [50]. No studies on secondary testing of pathogen reduced units have been published to date. The small amount of information on three studies of secondary testing of a total of 14,551 LVDS units was recently reviewed by Delage and Bernier [47] and is shown in Table 7. The overall contamination rate, 439 (95% CI 217–904) per million, is remarkably similar to that found in studies not using LVDS of 386 (95% CI 314–474) per million (Figure 3) [13]. Therefore, at this point in time, there is insufficient evidence to show that platelet units released using pathogen reduction and primary culture LVDS strategies have lower rates of bacterial contamination than those tested by previous primary culture protocols.

## 8. The Consequences of Transfusing Contaminated Products

Reactions to transfusion of bacterially contaminated platelet products, referred to as septic reactions, are characterized by any or all of these features—fever, rigors, dyspnea, nausea, vomiting, tachycardia, hypotension and shock occurring during or shortly after transfusion. Definitive diagnosis of a septic reaction is made by culture of the same bacterial species from the platelet unit involved and from a blood culture from the recipient obtained during the reaction. This limits the attribution of septic reactions to platelet transfusions if platelet units are not cultured or if appropriate blood cultures are not obtained from the patient.

Another obvious limitation of septic reaction data is failure to report reactions. Transfusion reaction surveillance programs can be active or passive. Active clinical surveillance programs use dedicated hemovigilance officers to monitor transfused patients and check that appropriate investigation and reporting occurs. Active bacteriologic surveillance by at-issue culture is another form of active surveillance, with analysis of 22 septic reactions showing that 18 (81.9%) were recognized by caregivers but only 8 (36.4%) were reported to the transfusion service, while the remaining 4 cases (18.2%) were not recognized as septic reactions [13]. Passive surveillance relies on caregivers recognizing, investigating and reporting transfusion reactions consistent with septic reactions. The incidence of septic reactions by active surveillance to apheresis and prepooled platelet products released as negative after primary culture has been remarkably constant over time, ranging from 36 to 50 cases per million transfusions; in contrast, the incidence was 5- to 10-fold lower, ranging from 1 to 10 cases per million transfusions, with passive surveillance systems [26]. The limitations of passive surveillance are illustrated by a passive surveillance report, requiring that the same bacterial species be isolated from the platelet unit and the patient’s blood culture; of 856 reported cases of suspected platelet transfusion associated sepsis investigated from 2012 through 2019 by the UK Serious Hazards of Transfusion (SHOT) group, only one case was determined to be a septic reaction [51].

**Figure 4:**
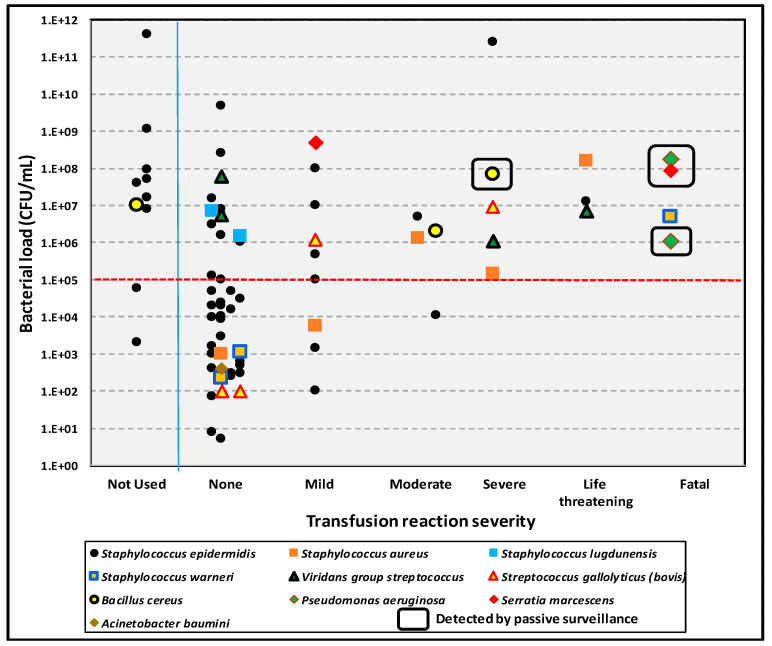
Plot of bacterial species and loads of bacterially contaminated platelet units against severity of transfusion reactions, University Hospitals Cleveland Medical Center, 1991–2017 [13]. During this period, 80 contaminated units were detected and 70 of these were transfused, resulting in 25 septic reactions. Only 4 septic reactions were reported to the transfusion service by passive surveillance.

The consequences of transfusing bacterially contaminated platelet products have been documented in a long-term, single institution study of 126,052 units cultured at time of issue for transfusion [13]. Septic reactions occurred in 25 recipients of 69 transfused, contaminated units, with reactions and severity of reactions related to organism load and virulence of the bacterial species involved. More virulent species included gram-negative bacilli, *Staphylococcus aureus*, *Staphylococcus lugdunensis*, and *Bacillus cereus*. Septic reactions rates due to less versus more virulent organisms were 135 and 40 per million, respectively, with no significant differences in the severity of septic reactions, time of onset of septic reactions, and mean bacterial loads (1.04 × 10^10^ vs. 1.82 × 10^7^ CFU/mL; *p* = 0.17) between units contaminated with less versus more virulent organisms. Bacterial load showed a much more significant correlation with septic reactions, with 21/35 (60.0%) of transfusions with bacterial loads of >10^5^ CFU/mL resulting in septic reactions versus 4/34 (11.8%) with bacterial loads of ≤10^5^ CFU/mL (*p* < 0.001) (Figure 4).

The sensitivity and specificity of various definitions of septic reactions is another issue confounding data on this topic. Active bacteriologic surveillance over a 7-year period documented that 20 of 51,440 platelet units transfused (0.004%; 389 per million) were bacterially contaminated and resulted in 5 septic reactions occurring 9 to 24 h posttransfusion; none of these septic reactions had been reported by passive surveillance [50]. Septic reactions occurred only in neutropenic patients transfused with high bacterial loads. During the study period a total of 284 transfusion reactions (0.55%) were reported to the transfusion service by passive surveillance; none of these patients had received contaminated platelet products. However, 6 to 93 (2.1–32.7%) of these 284 reactions met one or more septic reaction criteria, and the sensitivity of these criteria varied from 5.1% to 45.5%. These results document the failure of passive surveillance to detect septic reactions and the lack of specificity of their criteria. These findings highlight the limitations of reported national septic reaction data based on passive surveillance and the limited value of meta-analyses performed on passive surveillance reports [52].

Despite the many limitations on the accuracy and value of data on septic reactions, severe reactions that occur during transfusion of a platelet product are usually recognized and reported, particularly if the patient succumbs to the sepsis. The FDA publishes an annual report on Fatalities Reported to FDA Following Blood Collection and Transfusion, which includes data on fatalities associated with transfusion of platelet products, with the most recent report published in 2023 covering up to fiscal year 2021 [53]. A summary of these reports for fiscal years 2015 through 2021 is shown in Table 8. A total of 20 fatalities were reported, with 18 associated with higher virulence species. Platelet types involved were 17 apheresis units from 15 apheresis collections, 2 pathogen reduced apheresis units and one prestorage pooled unit. With approximately 2 million platelet transfusions a year in the US, 20 fatalities over 7 years represent a fatality rate of 1.4 per million transfusions (1:700,000 transfusions).

## 9. Recent Cases of Polymicrobial Contamination of Platelet Concentrates with a Likely Common Source

A recent investigation of a spate of platelet transfusion-related sepsis cases in the past five years has shed light on some of the limitations of current strategies to decrease the risk of bacterial contamination of platelet products. From 2018–2022, seven cases of polymicrobial contamination were reported to the FDA, and three of these cases resulted in patient fatalities [14,54,55,56]. Notably, these products contained the uncommon contaminant organisms *Acinetobacter calcoaceticus-baumanii* complex and *Staphylococcus saprophyticus*, as summarized in Table 9, despite a variety of pathogen control strategies utilized in the affected products. This series of cases importantly represented the first reports of pathogen-reduced platelet products being responsible for transfusion-related sepsis.

At the occurrence of the first two of these cases in 2018, the CDC Emerging Infections Network and Epidemic Information Exchange initiated a call for additional cases of sepsis [14], leading to an FDA safety communication in April 2019, updated in 2021 and 2022, encouraging reporting by facilities of platelet transfusion associated septic reactions caused by *Acinetobacter calcoaceticus-baumanii* complex and *S. saprophyticus*, with eventual inclusion of *Leclercia adecarboxylata* as well [57]. Initial conjectures for the etiology of sepsis-capable product after pathogen reduction centered on local environmental contamination after pathogen reduction, such as from a container leak [17]. However, it eventually became apparent after whole genome sequencing of 35 additionally identified isolates from platelet components as well as 34 isolates from 90 environmental samples from five states that outbreak clusters were readily identifiable for each of these bacterial species. Additionally, the presence of a novel *Acinetobacter calcoaceticus-baumanii* complex species at different times and locations strongly suggested a common contamination site upstream in the collection or production process. The investigators thus shifted their attention to the complex workflow of platelet components, performing detailed traceback and eventually identifying likely sources as platelet collection set manufacturing facilities in the Dominican Republic and Puerto Rico. Specimens collected from these manufacturing facilities clustered with the previously identified isolates, thereby corroborating the hypothesis (Table 10).

This study has multiple ramifications for the current trajectory of improving the safety of platelet transfusions. First, it is clear that pathogen reduction, at least in its current state, is by no means a guarantee for prevention of transfusion-related sepsis. Several mechanisms could be responsible for the findings in this study, ranging from spore-forming bacteria to biofilms or simply high initial inoculum, which would somewhat bypass the 7-log decrease in viable organisms seen with pathogen reduction. Further study into pathogen reduction resistance is thus warranted. It must also be noted that the true incidence of significant pathogen load in products is likely underestimated due to asymptomatic patients and failure to report septic reactions [50]. As an example, in the series reported by Kracalik et al., implicated platelet product co-components were transfused into three other patients without incident [14]. Even though measures taken by the FDA here were able to alert transfusion facilities of the possibility of these new contaminants, ultimately, additional effort will be required to prevent their occurrence.

## 10. Platelet Product Use in the US

The US Department of Health and Human Services’ National Blood Collection and Utilization Survey (NBCUS) has been conducted biennially since 1997, with findings for 2021 published in 2023 [44,58]. These reports document that 2,327,000 platelet units were distributed in 2021, with 2,175,000 units transfused, indicating that 152,000 units (6.5%) were not used. Transfused unit types were predominantly apheresis (2,011,000, 96.1%), with 80,000 (3.9%) whole-blood derived. Platelet concentrates were predominantly suspended in plasma (84%), with 16% suspended in plasma with platelet additive solution. Between 2019 and 2021, the proportion of transfusing facilities reporting use of pathogen-reduced platelet units increased from 13% to 60%. Almost half (48.5%) of apheresis units were transfused on day 4–5, with 41.4% transfused on day 1–3 and 10.1% on day 6–7.

Bacterial mitigation methods used on apheresis units were pathogen reduction (41.9%) and primary culture (58.1%). Primary culture methods were low volume culture (34.1%), 36-h LVDS (36.0%) and 48-h LVDS (29.8%). As low volume culture was discontinued at the end of September, 2021 to comply with the new FDA guidance recommendations, for the period January through September 2021, 42.0% of apheresis units transfused had been tested using low volume primary culture versus 60.8% by LVDS, indicating significant use of LVDS prior to its required implementation date of October 1, 2021. Secondary testing was rarely used, with secondary culture used on 9131 and rapid test used on 14,764 apheresis units, representing 1.1% and 1.8%, respectively, of apheresis units eligible for secondary testing to extend shelf-life. None of the secondary culture tests were positive, while 6 of 14,764 (406 per million) were positive by rapid test. Only 57 platelet units had reported transfusion associated bacterial contamination, a rate of 26 per million based on all platelet product transfusions.

Median cost of platelet products in 2021 was $567 per unit for leukocyte reduced apheresis units tested by primary culture, a 9% increase compared to cost in 2017 and 2019 of $516 per unit, reflecting in part the higher cost of LVDS units. Median cost in 2021 of pathogen reduced units was $660, an 8.6% increase over the cost in 2019 of $617 per unit. Based on these costs, the total cost of platelet units for 2021 was $1.4 billion, with $644 million spent on pathogen reduced and $767 million on primary culture tested units. The incremental cost over 2019 costs was $209 million—$140 million ($144 per unit) for pathogen reduced and $69 million ($51 per unit) for LVDS units. The cost of the 152,000 unused units was $92 million.

## 11. Alternatives to Room Temperature Storage

The advent of pathogen reduction technologies does not imply that there is no attempt at looking at other means of improving and increasing the safety of platelet components. At a time when there are frequent blood component shortages, in particular platelet products, mechanisms for saving platelet units when collections increase to be used at a later time have become increasingly important. Some of these approaches include establishing cryopreservation or use of low temperatures and freezing to save platelets for longer shelf-life and later use [59]. These approaches have also been thought of as alternatives to decrease the risk of bacterial contamination of platelet units. Currently, platelet units, regardless of type of collection, are kept at room temperature with gentle agitation in order to maximize viability and time in circulation once transfused [60]. However, this approach has disadvantages, among which are limited storage duration as noted earlier, risk of bacterial contamination and growth, and higher costs [61]. As a result, it is thought that cold-stored platelets can improve upon those disadvantages found with room temperature stored platelets [62]. This is despite initial reports that indicated that cold-stored platelets had a more rapid clearance from circulation and lower response to agonists [63,64] but this may not be the case since those phenotypic changes may have been overestimated and are partly due to impaired taurine and purine metabolism [65]. This is the driving force behind the development of systems that improve platelet longevity and increased research into cold-storage of platelet concentrates or freezing of units after collection for use at times of low platelet product inventories [66].

Cold-stored platelet concentrates are maintained at temperatures of 1–6 °C until needed. Platelet concentrates stored in this manner have been reported to have greater hemostatic capability compared to platelets maintained at room temperature [67]; this has also been shown to be the case in animal models [68]. In part, this capability appears to be due to lower temperature slowing down platelets’ metabolism, resulting in priming or greater aggregation once in vivo [67]. This greater hemostatic capacity also results from production of a higher number of large platelet-derived extracellular vesicles that decrease the time needed for clot formation [69]. Furthermore, this greater state of activation is a direct response to observed increases in cytosolic calcium released from the endoplasmic reticulum [70]. Cold storage also inhibits GPIb-α mediated apoptotic signals thus improving physiological availability of these platelets in circulation [71]. Likewise, cold-stored platelets remain functional and able to aggregate even after 21 days of storage, but this is reduced if cold storage is delayed [72]. All of these benefits support the use of such platelet products in clinical practice.

Use of these cold-stored platelet units, however, require implementation of mechanisms at the donation facility which prepare the units so that they are maintained at a constant temperature, go through transportation to the destination facility and that the latter is able to keep storage under the same conditions. Nevertheless, implementation and usage of these units may not decrease the platelet product wastage at the destination facility [73]. Use of sodium citrate as a buffer in the platelet additive solution maintains the activity of cold stored platelet concentrates without sacrificing yield [74]. Perhaps the one variable to be considered in these platelet units is that body mass index (BMI) influences the degree of activation since donors with higher BMI had platelets with a lower activation capability and lower overall platelet numbers after cold storage and subsequent transfusion [75].

Platelet concentrates frozen in dimethyl sulfoxide (DMSO) have been shown to be similar in quality to other platelet components, as reported by the two-decades long experience of the military with no data indicative of higher rates of bacterial contamination [76]. Similar successes have been obtained when freezing platelet concentrates derived from buffy coats in additive solution and DMSO [77]. Just as shown for cold-stored units, in vitro and in vivo studies have shown these platelets to be primed for activity and to have a hypercoagulable phenotype upon thrombin activation under all experimental assays [78]. Notably, most recent studies indicate that cryopreserved and cold platelets display a pro-coagulant profile that produces a rapid hemostatic response, which is needed in actively bleeding patients [79]. Despite this, it should be expected that collected platelets in a frozen unit are heterogeneous and thus contain subpopulations that can be more sensitive to freezing [80]. In light of this, approaches that reduce levels of calcium, which is known to cause cryopreservation-induced platelet damage, through the use of a calcium chelator prior to preservation to limit the amount of damage that platelets undergo while freezing [81]. Furthermore, even when frozen just prior to the unit’s expiration, the quality of platelets does not seem significantly compromised compared to platelet units’ activity early during their storage period [82]. Perhaps the only caveat is that thawing of frozen platelet units removes up to half of platelets from the unit, but those remaining are functionally viable regardless of duration of cryopreservation [83]. Taken together these data support the use of both cold-stored and frozen platelet units and indicate that they do not result in less functional platelets, with the added benefit of an apparently decreased risk of bacterial contamination.

## 12. Conclusions

Strategies introduced over the past two decades to limit bacterial contamination of platelet products have included:

- Optimizing the skin preparation technique of donors
- Diversion of the initial 15 to 30 mL of the blood draw
- Ensuring sterility and integrity of materials and methods used to process blood components
- Changing supply from whole-blood derived to apheresis units
- Primary culture performed at 24 h using low volume (4–8 mL) culture in aerobic bottles of apheresis collections and whole-blood derived pools
- Secondary testing using low volume (4–8 mL) culture in aerobic bottles of apheresis collections and whole-blood derived pools
- Secondary rapid testing within 24 h of use of apheresis collections and whole-blood derived pools
- LVDS primary culture performed at 36–48 h using 16 mL culture volume, divided between aerobic and anaerobic bottles, of split apheresis units and whole-blood derived pools
- Pathogen reduction without testing for bacterial contamination

How effective have these measures been? Unfortunately, no studies subsequent to the PASSPORT study have been performed with a clear criterion for success set by the FDA based on secondary culture, and this principle has not been applied to the strategies included in the recent FDA guidance document. However, the following generalizations can be made about these measures:

- All these strategies have reduced, but not eliminated, transfusion transmitted bacterial sepsis.
- Secondary testing on day 3 or day 4 has been very effective in preventing transfusion of contamination missed by primary culture, but is unfortunately hardly used due to logistic and cost issues [44,58].
- The predominant bacterial mitigation methods now used are LVDS and pathogen reduction, with high incremental costs for both these strategies (over $200 million per year in the US) [44].
- Pathogen reduction was considered to be a highly effective strategy, but four recent cases of severe septic reactions, two fatal, have challenged this view [14], and no systematic culture studies on pathogen reduced units at time of use or outdate have been performed.
- LVDS has been in use since 2011 and has been documented to reduce septic reactions compared to no testing, but absolute evidence that it is better than low volume primary testing is lacking, with the little secondary culture data available (Table 4) showing contamination rates (415 per million; 95% CI 195–903) comparable to those found by secondary testing following low volume primary culture (386 per million; 95% CI 314–474) (Figure 3).
- Use of anaerobic culture bottles is an issue as it results in wastage of units contaminated with anaerobes such as *Cutibacterium* (*Propionibacterium*) *acnes* that are of no clinical significance.
- The high incremental cost ($209 million in the US in 2021—$140 million for pathogen reduced and $69 million for LVDS units—is hard to justify and better evidence of efficacy of these strategies is needed.
- Performing secondary culture studies to assess the efficacy of pathogen reduction and LVDS would cost a fraction of the over $200 million per year incremental costs of these new strategies.
- Another issue that needs to be addressed in the high outdate rate for platelets, with the cost in 2021 in the US of 152,000 unused units being $92 million [58].

Are any additional steps needed to further reduce the risk? Yes, there are, but cost and logistic issues limit use of secondary testing as discussed above. Better evidence of the efficacy of LVDS would be very helpful in determining the cost-effectiveness of LVDS platelet products and whether secondary testing would be more cost effective than LVDS. Cold storage and frozen platelet concentrates could be very effective strategies, with studies showing that platelet function is maintained under conditions that avoid storage at 20–24 °C storage that allows bacterial proliferation.

Bacterial contamination of platelet units resulting in sepsis in recipients continues to challenge us. Recent cases of polymicrobial contamination of platelet concentrate collecting systems presented an unexpected source of contamination, and the mechanism of how these organisms avoided inactivation by amotosalen is not known at this time. A coordinated national surveillance system to report unusual bacterial species cultured from primary cultures and determine their clonality would be very helpful in detecting common sources as shown by the investigation of the polymicrobial contamination outbreak.

## Figures and Tables

**Table 1:** Sources of bacterial contamination of platelet units with examples of typical bacterial species associated with each source.

Source	Examples
**Bacteria originating from skin of donors**	*Staphylococcus aureus* *Staphylococcus epidermidis* Other coagulase-negative staphylococci *Corynebacterium species* *Cutibacterium* (*Propionebacterium*) *acnes* *Clostridium perfringens*
**Transient bacteremia with mucous membrane microbiota**	Viridans group streptococci *Enterococcus faecalis*
**Transient bacteremia associated with gastrointestinal malignancy**	*Streptococcus gallolyticus* (*bovis*)
**Bacteremia associated with acute gastrointestinal disease**	*Listeria monocytogenes* *Yersinia enterocolitica* *Enterobacterales* (e.g., *Escherichia coli* and *Klebsiella pneumoniae*)
**Bacteremia associated with chronic osteomyelitis**	*Salmonella species*
**Contaminated reagents in platelet collection kits**	*Enterobacterales* (e.g., *Leclercia adecarboxylata*) Coagulase-negative staphylococci (e.g., *Staphylococcus saprophyticus*) *Acinetobacter species*
**Environmental contaminants**	*Enterobacterales* (e.g., *Serratia marcescens*) *Bacillus species* *Acinetobacter species* *Pseudomonas species*
**Retrograde contamination**	Bacterial species responsible for bacteremia in a patient receiving a platelet transfusion, e.g., *Enterococcus faecalis* and coagulase-negative staphylococci

**Table 2:** Estimates of time one organism in a 350 mL platelet unit will reach 10^5^ CFU/mL based on bacterial growth rates in platelet components during storage. Bacterial growth rates were based on data presented by Walker at al. [25].

Bacterial Species	Median Lag Time (Hours)	Median Doubling Time (Hours)	Time in Log Phase to Reach 10^5^ CFU/mL (Days) *	Time to Reach 10^5^ CFU/mL (Lag + Log Phases) (Days)
*Bacillus cereus*	5	1.5	1.6	1.8
*Enterococcus faecalis*	8	3.0	3.1	3.5
*Escherichia coli*	6	1.3	1.4	1.6
*Klebsiella oxytoca*	8	1.2	1.3	1.6
*Klebsiella pneumoniae*	8	1.2	1.3	1.6
*Lactococcus garvieae*	5	1.4	1.5	1.7
*Serratia liquefaciens*	10	1.8	1.9	2.3
*Serratia marcescens*	10	1.2	1.3	1.7
*Staphylococcus aureus*	8	2.0	2.1	2.4
*Staphylococcus epidermidis*	18	4.0	4.2	4.9

* A single bacterium in a 350 mL platelet unit can reach a bacterial load of 10^5^ CFU/mL after 25 doublings in log growth phase.

**Table 3:** Primary culture strategies for limiting bacterial contamination of apheresis collections and units, and pre-storage whole blood derived pools under FDA guidance implemented in October 2021 [11]. Guidance specifies that times in hours are defined as exact time after collection or sampling, while times in days are defined as any time on day specified. Another strategy allowed by this FDA guidance is pathogen reduction of apheresis units treated with an FDA-cleared method performed within 24 h of collection, with treated units having a shelf-life of 5 days.

Primary Culture Volume	Platelet Unit Types	Primary Culture Sample Time (Hours)	Primary Culture Release Time (Hours)	Expiration Time (Days)	Secondary Test Method	Secondary Test Sample Time (Days)	Secondary Culture Release Time (Hours)	Expiration Time after Secondary Test (Days)
**Single-step strategies**								
≥8 mL AER ≥8 mL ANA	APH collections APH split units WBD pools	≥24	12	3	NA	NA	NA	NA
≥8 mL AER ≥8 mL ANA	APH split units WBD pools	≥36	12	5	NA	NA	NA	NA
≥8 mL AER ≥8 mL ANA	APH split units	≥48	12	7	NA	NA	NA	NA
**Two-step strategies**								
≥8 mL AER ≥8 mL ANA	APH collections APH split units WBD pools	≥24	12	3	Culture ≥8 mL AER	3	User defined	5
≥8 mL AER ≥8 mL ANA	APH collections APH split units WBD pools	≥24	12	3	Culture ≥8 mL AER ≥8 mL ANA	4	12	7
≥8 mL AER ≥8 mL ANA	APH split units	≥36	12	5	Culture ≥8 mL AER	3	User defined	7
≥8 mL AER ≥8 mL ANA	APH split units	≥36	12	5	Culture ≥8 mL AER ≥8 mL ANA	4	12	7
≥8 mL AER ≥8 mL ANA	APH collections APH split units WBD pools	≥24	12	3	Rapid test	Within 24 h of use	NA	In PAS: 5 In plasma: 7
≥8 mL AER ≥8 mL ANA	APH split units WBD pools	≥36	12	5	Rapid test	Within 24 h of use	NA	In plasma: 7

Guidance specifies that times in hours are defined as exact time after collection or sampling, while times in days are defined as any time on day specified. AER, aerobic culture bottle cleared for platelet product culture. ANA, anaerobic culture bottle cleared for platelet product culture. APH, apheresis platelet product. NA, not applicable. PAS, platelet additive solution. WBD, whole blood derived platelet product.

**Table 4:** Strategies for limiting bacterial contamination of single and post-storage pooled whole-blood derived units under FDA guidance implemented in October 2021 [11].

Product	Strategy	Primary Culture Minimum Volume per Bottle (Bottle Type)	Incubation Time before Release (Hours)	Shelf Life (Days)
**Single units**	Rapid testing of single WBD units within 24 h of transfusion	NA	NA	5
	Primary culture ≥ 24 h after collection	Largest practical volume (aerobic)	12	3 ^a^
	Primary culture ≥ 36 h after collection	Largest practical volume (aerobic)	12	5
**Post-storage pools**	Rapid testing of post-storage pools at time of pooling ^b^	NA	NA	5

Guidance specifies that times in hours are defined as exact time after collection or sampling, while times in days are defined as any time on day specified. ^a^ Post-storage whole-blood derived pools expire 4 h after pooling. ^b^ Can be extended to 5 days with rapid testing.

**Table 5:** Sensitivity (Limit of Detection) of PGD*prime* used to test apheresis platelet concentrates in plasma [36].

Organism *	Limit of Detection (CFU/mL)
*Bacillus cereus*	2.7 × 10^4^
*Clostridium perfringens* ATCC 13124	2.4 × 10^4^
*Escherichia coli*	5.6 × 10^4^
*Klebsiella aerogenes*	3.3 × 10^4^
*Klebsiella pneumoniae*	6.1 × 10^4^
*Pseudomonas aeruginosa*	2.6 × 10^3^
*Serratia marcescens* ATCC 43862	2.5 × 10^6^
*Staphylococcus aureus*	2.1 × 10^3^
*Staphylococcus epidermidis*	1.9 × 10^3^
*Streptococcus agalactiae*	1.6 × 10^5^
*Streptococcus oralis*	2.5 × 10^6^
*Acinetobacter baumannii* ATCC 19606	8.8 × 10^4^

* Unless otherwise noted, bacterial strains were isolates from blood cultures or recovered from platelet contamination events.

**Table 6:** Confirmed positive cultures from 960,470 apheresis units tested with LVDS protocol [12]. Split apheresis platelet units were sampled at 36 to 48 h after donation and 8 mL inoculated into each of aerobic and anaerobic BacT/ALERT culture bottles. Table shows details of the bacterial species or groups isolated, with bottle type and time to detection.

Bacterial Species/Group	No. (%) Isolated	Bottle Type: Aerobic Only/Anaerobic Only/Both	Time to Detection Range (Hours)
*Staphylococcus aureus*	8 (3.8)	0/0/8	6–14
Coagulase negative staphylococci	20 (9.6)	5/7/8	11–42
*Staphylococcus saccharolyticus*	17 (8.2)	0/15/2	51–70
*Cutibacterium* (*Propionebacterium*) *acnes*	74 (35.6)	0/63/11	56–135
Viridans group streptococci	51 (24.5)	9/12/30	8–41
*Streptococcus gallolyticus* (*bovis*)	7 (3.4)	1/1/5	8–34
*Enterobacterales*	9 (4.3)	0/1/9	3–16
*Listeria monocytogenes*	4 (2.1)	0/1/3	14–20
Other	18 (8.6)	3/13/2	6–92
**Totals**	**208** (100)	**17/113/78**	**3–135**
**Totals excluding strict anaerobes ^a^**	**111** (53.4)	**17/22/78**	**3–42**

^a^ Cutibacterium (Propionebacterium) species, Staphylococcus saccharolyticus and Peptostreptococcus species.

**Table 7:** Secondary testing of LVDS platelet units [47].

Study	No. Tested	No. Positive	Rate per Million	95% CI
NHSBT, UK	6014	1	166	40–926
Héma-Québec, Canada	4536	1	220	53–1227
Canadian Blood Services	5400	5	926	408–2159
Total	15,950	7	439	217–904

**Table 8:** Fatalities Reported to FDA Following Platelet Transfusions, fiscal years 2015 through 2021 [53]. Unless indicated otherwise indicated, implicated platelet units were primary culture negative apheresis units.

	Fiscal Year ^a^							
Organism	2015	2016	2017	2018	2019	2020	2021	Total
*Acinetobacter* spp.				1				**1**
*Clostridium perfringens*			2 (co-comp)					**2**
*Corynebacterium striatum*							1	**1**
*Enterobacter aerogenes*		1						**1**
*Enterococcus faecium*	1							**1**
*Escherichia coli*							2 (co-comp)	**2**
*Klebsiella pneumoniae*			1					**1**
*Serratia marcescens*					1 (pool)			**1**
*Staphylococcus aureus*	3			2			1	**6**
*Staphylococcus epidermidis* or other CoNS	1		1					**2**
Polymicrobial ^b^						1 (PR) ^b^	1 (PR) ^c^	**2**
**Total**	**5**	**1**	**4**	**3**	**1**	**1**	**5**	**20**

Co-comp = co-components from the same apheresis collection. Pool = pooled apheresis unit. PR = pathogen reduced unit. ^a^ Fiscal year is October 1 of the prior year to September 30 of the current year. ^b^ FY2020 case of polymicrobial contamination involved *Acinetobacter* sp., *Leclercia adecarboxylata* and *Staphylococcus saprophyticus*. ^c^ FY2021 case involved *Bacillus* species (not *Bacillus anthracis*), *Acinetobacter baumannii* complex, *Leclercia adecarboxylata* and *Staphylococcus saprophyticus*.

**Table 9:** Characteristics of polymicrobial contaminated sepsis cases associated with apheresis platelet concentrates collected using Amicus collection sets, with platelets suspended in platelet additive solution. Adapted from Kracalik et al. [14].

Patient Characteristics							
Case number	A	B	C ^c^	D ^c^	E	F	G
Year	2018	2018	2018	2018	2020	2021	2021
State	California	Utah	Connecticut	Connecticut	North Carolina	Ohio	Virginia
Fatal outcome	−	+	−	−	+	+	−
Bacterial control strategy ^a^	PR	PC	PC + SR	PC + SR	PR	PR	PR
**Post-transfusion culture source ^b^**							
*Acinetobacter calcoaceticus-baumanii* complex	BP	BP	BP	BP	BP	B	−
*Staphylococcus saprophyticus*	P	−	BP	BP	BP	B	P
*Leclercia adecarboxylata*	−	−	−	−	BP	P	P
*Enterobacter* species	−	−	−	−	−	−	BP
*Bacillus* species	−	−	−	−	−	P	−

^a^ Bacterial control strategy: PC = primary culture, PR = pathogen reduction, SR = secondary rapid test. ^b^ Post-transfusion culture sources: B = blood, P = platelet product. ^c^ Patients C and D received co-components from the same platelet product collection.

**Table 10:** Sources and clustering of *Acinetobacter calcoaceticus–baumannii* complex (ACBC) and *Staphylococcus saprophyticus* associated with common source contaminated platelet product cases by state and origin of isolates (clinical, platelet product or environment). Data adapted from Kracalik et al. [14]. Isolates were obtained from 7 patients and/or platelet units associated with septic transfusion reactions (noted in Table 9 as cases A–G), by primary bacterial culture screening of interdicted units, and from environmental sampling of surfaces such as equipment used to store platelet components in blood establishments and healthcare facilities. Environmental sampling also included platelet collection sets and associated solutions (saline and anticoagulant) collected in two platelet collection set manufacturing facilities located in Puerto Rico and the Dominican Republic.

Geographic Source	Number of Isolates of Each Organism Group by Specimen Source ^a^		
	Patient	Platelet Product	Environment
**ACBC Cluster 1: ** ***Acinetobacter calcoaceticus-baumannii* complex** **, Novel (n = 21)**			
California	1 (A)	1 (A)	2
Connecticut	2 (C, D)	3 (C, D)	1
Missouri		1	
North Carolina		3 (E)	2
Utah	1 (B)	1 (B)	3
**ACBC Cluster 2: *Acinetobacter seifertii* (n = 15)**			
California		2	9
Connecticut			1
North Carolina	1 (E)		
Ohio		1 (F)	
Utah			1
**ACBC not in Clusters 1 or 2 (n = 35)**			
Dominican Republic			12
Puerto Rico			23
***Staphylococcus saprophyticus *** **Cluster A (n = 21)**			
California		6 (A)	6
Connecticut	1 (D)	2 (D)	
Massachusetts		1	
Missouri		1	
North Carolina		2	1
Ohio		1 (F)	
***Staphylococcus saprophyticus* Cluster B (n = 40)**			
AZ		2	
California		1	1
Connecticut	1 (C)	2 (C)	2
Massachusetts		1	
Maryland		1	
North Carolina		1	1
NJ		1 (G)	
NY		1	
Ohio	1 (F)	3 (F)	
Puerto Rico			21
***Staphylococcus saprophyticus* Cluster C (n = 3)**			
North Carolina	1 (E)	1 (E)	
Ohio		1	
***Staphylococcus saprophyticus* not in Clusters A, B or C (n = 28)**			
Dominican Republic			15
Puerto Rico			13

^a^ Letters in parentheses refer to patient case numbers in Table 9. Platelet product isolates without patient case numbers were recovered from positive primary cultures of interdicted platelet units. ACBC, *Acinetobacter calcoaceticus-baumannii* complex.
